# Ethylene Biosynthesis Dynamics and Genome-Wide Characterization of the ACS and ACO Gene Families During Fruit Development in ‘Kuerle Xiangli’ (*Pyrus sinkiangensis* Yu)

**DOI:** 10.3390/biology15191769

**Published:** 2026-10-04

**Authors:** Tingting Fan, Deen Zhang, Wenjie Li, Zhihua Guo, Xiaoyan Lu

**Affiliations:** 1 Department of Horticulture, College of Agriculture, Shihezi University, Shihezi 832003, China; 2 Key Laboratory of Special Fruits & Vegetables Cultivation Physiology and Germplasm Resources Utilization of Xinjiang Production and Construction Group, Shihezi 832003, China; 3 Workstation for Academician Li Tianlai, Shihezi University, Shihezi 832003, China

**Keywords:** ethylene biosynthesis, *ACS* gene family, *ACO* gene family, fruit ripening, gene expression

## Abstract

**Background:** Fruit ripening in ‘Kuerle Xiangli’ (*Pyrus sinkiangensis* Yu) is a complex physiological process orchestrated by ethylene; however, the molecular mechanisms governing ethylene biosynthesis during fruit development to ripening remain incompletely understood. **Methods:** In this study, we systematically characterized ethylene production, fruit quality parameters, and the ACS (1-aminocyclopropane-1-carboxylic acid synthase) and ACO (ACC oxidase) gene families in ‘Kuerle Xiangli’ across nine developmental stages from 30 to 150 days after full bloom (DAFB). **Results:** Ethylene production remained suppressed during fruit growth and expansion but exhibited a massive climacteric burst at the ripening stage (150 DAFB). Soluble solids content peaked at 120 DAFB (16.2%), whereas fruit firmness declined significantly from 135 to 150 DAFB, indicating a temporal asynchrony between sugar accumulation and cell wall disassembly. A total of 12 *PsinACS* and 5 *PsinACO* genes were identified from the genome (*Pyrus sinkiangensis* Yu). Phylogenetic analysis classified the PsinACS proteins into four groups (I–IV) and the PsinACO proteins into two groups (B and C), with Group I ACS members clustering with well-characterized ripening-associated orthologs. Conserved motif and gene structure analyses revealed high conservation within each phylogenetic group. Gene duplication analysis indicated that most *PsinACS* and *PsinACO* genes originated from whole-genome duplication or segmental duplication events, with only a few arising from proximal or tandem duplications. Synteny analysis demonstrated strong evolutionary conservation across Rosaceae species, and Ka/Ks ratios (<1) indicated that both gene families have undergone strong purifying selection. Expression profiling by qRT-PCR revealed distinct patterns: *PsinACS2*, *PsinACS3* and *PsinACS4* were highly expressed in early fruitlets (30 DAFB), and eight *ACS* genes (*PsinACS1, PsinACS5–9*, *PsinACS11–12*) and *PsinACO1* showed maximal expression at 150 DAFB, coinciding with the ethylene climacteric burst. **Conclusions:** Taken together, combined with phylogenetic tree analysis, indicates that *PsinACS1* and *PsinACO1* are crucial genes in ethylene biosynthesis that positively regulate fruit ripening. These results provide a comprehensive characterization of the *ACS* and *ACO* gene families in *P. sinkiangensis* and reveal a coordinated transcriptional network that governs ethylene biosynthesis during pear fruit ripening.

## Simple Summary

Fruit ripening in pear is a complex developmental process involving coordinated changes in ethylene production, fruit texture, soluble solids accumulation, and the expression of ethylene biosynthesis genes. *Pyrus sinkiangensis* (Kuerle Xiangli), a characteristic and economically important fruit cultivar in Xinjiang, China, exhibits typical climacteric ripening characteristics. A comprehensive analysis of these gene families and their developmental expression patterns in ‘Kuerle Xiangli’ remains limited. In this study, we investigated fruit development and ripening across nine stages from 30 to 150 days after full bloom. We characterized changes in ethylene production and performed a genome-wide analysis of the *ACS* and *ACO* gene families.

## 1. Introduction

Fruit ripening represents a highly coordinated, genetically programmed developmental process that culminates in dramatic changes in color, texture, flavor, aroma, and nutritional composition [1,2]. Ethylene serves as the master hormonal regulator in climacteric fruits, governing a cascade of downstream transcriptional and biochemical events that drive the ripening [3]. A recent review of pear fruit quality further highlights the central role of ethylene in pear ripening, softening and quality regulation [4].

The ethylene biosynthetic pathway in higher plants is well established. Ethylene is synthesized from S-adenosyl-L-methionine (SAM), which is first converted to 1-aminocyclopropane-1-carboxylic acid (ACC) by ACC synthase (ACS), and subsequently oxidized to ethylene by ACC oxidase (ACO) [5]. *ACS* and *ACO* gene families have been extensively characterized in many fruit species. In tomato, a model climacteric fruit, at least nine *ACS* genes *(LEACS1A, LEACS1B*, and *LEACS2* to *8*) and five *ACO* genes (*LEACO1* to *5*) were identified [6]. In pear, a comprehensive genome-wide analysis of the *ACS* and *ACO* gene families was first reported in *Pyrus ussuriensis*, identifying 13 *ACS* and 11 *ACO* genes, with 6 *ACS* and 4 *ACO* genes displaying ripening-associated expression patterns [7]. Within the Rosaceae, apple and peach are climacteric fruits and share greater similarity with pear in ripening physiology. The suppressed expression of *PpACS1* is responsible for extremely low ethylene production and the non-softening phenotype in stony hard peach fruit, demonstrating the decisive role of *ACS1* in peach climacteric ripening [8], while banana *MaACS1* acts as the core regulator triggering climacteric ethylene burst, with post-transcriptional restriction of *ACO* activity further modulating ethylene peak characteristic [9]. In pear, ripening-associated ethylene synthesis genes have been revealed by comparative transcriptome analyses of five main pear species; the expression of two *ACS* related genes was especially high at the stage of fruit maturation [10]. The climacteric cultivar ‘Nanguoli’ exhibits intensive ethylene production and rapid flesh softening during fruit ripening, accompanied by a significantly elevated expression of *ACS* genes at the mature stage [10,11], and the ACC synthase gene *PpACS1b* is regulated during late fruit development and responds to salicylic acid in *Pyrus pyrifolia* [12]. These studies collectively indicate that *ACS* and *ACO* gene families exhibit species-specific variations in gene number, phylogenetic distribution, and expression dynamics, underscoring the need for species-specific characterization to understand the molecular basis of ripening regulation.

In climacteric fruits, ethylene biosynthesis operates through two distinct regulatory systems: system I, which produces low levels of auto-inhibitory ethylene during growth and early fruit development, and system II, which generates high levels of auto-catalytic ethylene at the onset of ripening through the transcriptional activation of specific *ACS* and *ACO* genes [13]. The transition from system I to system II ethylene production represents a pivotal developmental switch that is tightly controlled by a hierarchical network of transcription factors, including MADS-box [14], NAC [15], and ethylene response factor (ERF) family members [16]. The release of the pear genome sequences for both Chinese white pear (*Pyrus bretschneideri*) [17] and ‘Kuerle Xiangli’ (*Pyrus sinkiangensis* Yu) [18] has facilitated the genome-wide identification of gene families involved in ethylene biosynthesis and signaling. Recently, the transcription factor *PbrERF114* was demonstrated to directly bind the promoter of *PbrACS3* and activate its expression, while also inducing *PbrERF24*, which in turn activates *PbrACO1* and *PbrACS3*, forming a transcriptional cascade that amplifies ethylene synthesis during pear fruit ripening [19]. Additionally, the sucrose-responsive transcription factor *PuWRKY31* has been shown to directly bind the promoters of *PuACS1a* and *PuACO1* to promote ethylene production in pear [20].

‘Kuerle Xiangli’ (*Pyrus sinkiangensis* Yu) is a premier pear cultivar originating from the Xinjiang region of China, renowned for its unique aromatic flavor, crisp texture, and high commercial value [20]. While genome-wide analyses of *ACS* and *ACO* genes have been reported in *P. ussuriensis* [7], a comprehensive genome-wide identification and systematic characterization of the *ACS* and *ACO* gene families in *P. sinkiangensis* is still lacking, and their expression dynamics in relation to ethylene emission across fruit developmental stages remain largely undefined. Furthermore, the correlation between ethylene biosynthesis gene expression and key fruit quality parameters, such as soluble solids content and firmness, during the transition from fruit development to ripening in ‘Kuerle Xiangli’ has yet to be established.

In the present study, we aimed to characterize the dynamics of ethylene production and changes in fruit quality parameters (SSC and firmness) across nine developmental stages from 30 to 150 DAFB in ‘Kuerle Xiangli’. *ACS* and *ACO* gene families at the genome-wide level were identified and comprehensively analyzed, including their phylogenetic relationships, conserved motifs, gene structures, chromosomal distribution, and duplication patterns. Moreover, the expression patterns of all *PsinACS* and *PsinACO* genes during fruit development and ripening using qRT-PCR determine which *ACS*/*ACO* members are the primary contributors to the climacteric ethylene burst during pear fruit ripening. This work provides a systematic foundation for understanding the molecular basis of ethylene biosynthesis in pear and offers candidate genes for future functional validation and molecular breeding aimed at improving fruit quality and storability.

## 2. Materials and Methods

### 2.1. Plant Materials

‘Kuerle Xiangli’ pear trees (*Pyrus sinkiangensis* Yu) were grown at the Second Division Institute of Agricultural Sciences, Xinjiang Production and Construction Corps, Xinjiang, China. The mesocarp tissues (flesh of Korla fragrant pear) were collected every 15 days from 30 days after full blooming (DAFB) to fruit ripening, and were collected at 30, 45, 60, 75, 90,105, 120, 135 and 150 days after full bloom (DAFB). For each sampling stage, fruits were randomly harvested from different trees within the same orchard block, and three biological replicates were prepared, each consisting of at least five fruits. Fruit flesh (mesocarp) was collected for gene expression and quality analyses, snap-frozen in liquid nitrogen, and stored at −80 °C.

### 2.2. Measurement of Ethylene

For ethylene measurement, fruits were sampled at each of the nine developmental stages (30, 45, 60, 75, 90, 105, 120, 135 and 150 DAFB). At each stage, three fruits were sealed together in a 2 L airtight jar to constitute one biological replicate, and three jars were prepared as three independent biological replicates for each sampling stage. Airtight jars were placed in a room under 25 °C environmental conditions. The ethylene production was measured using a gas chromatography system (GC2010, Shimadzu, Japan) with a 1 mL rheodyne injector (Gaoge, Shanghai, China) with a few modifications. The details were identical to those in a previous report [21].

### 2.3. Measurements of Fruit Firmness and Soluble Solids Content

Fruit firmness was detected using hardness tester (GY-3; Airuipu, Quzhou, China). Before measurement, the fruit epidermis at the equatorial position was peeled off to eliminate the interference of peel texture, and the penetration probe was vertically pressed into the fruit flesh at a constant speed. The resulting maximum resistance value was recorded as fruit firmness, expressed in the unit of kg/cm^2^. Soluble solids content (SSC) of fruit pulp was quantified using a portable digital refractometer (PAL-1; Atago, Tokyo, Japan). A small amount of fresh juice squeezed was dropped onto the detection prism of the refractometer, and the reading was directly recorded as the soluble solids concentration with the unit of percentage (%) according to a previous report [22].

### 2.4. Gene Identification

To identify the members of *ACS* and *ACO* gene families in pear, multiple database searches were performed. First, protein sequences encoded by candidate *ACS* and *ACO* genes were downloaded from the Pear Multiomics Database (PearMODB) (https://pearomics.njau.edu.cn/genome_data, accessed on 7 July 2026) using the gene IDs listed in Tables 1 and 2 of this paper [23], while protein sequences of other fruit tree species (apple, peach, orange, papaya, grape, tomato, and strawberry) were retrieved from the Phytozome database (https://phytozome.jgi.doe.gov/, accessed on 10 July 2026). The candidate *ACS*/*ACO* genes were initially identified by a BLASTP search against known ACS and ACO protein sequences. For the manual curation of candidate pear *ACS* and *ACO* genes, we retained only sequences containing the characteristic Pfam domains of *ACS* (PF00155) and *ACO* (PF14226; PF03171) to support gene annotation and confirm conserved functional domains [24]. The acquired web results with the output row format was reported. The isoelectric points, molecular weights, and lengths of the ACS and ACO proteins were obtained with the ExPasy website (https://web.expasy.org/protparam/, accessed on 10 July 2026).

### 2.5. Sequence and Phylogenetic Analyses of ACS and ACO Genes 

Multiple sequence alignment of the characterized ACS and ACO protein sequences was performed using Clustal W software (version 2.1). under the default parameter settings. After the null members were removed, the phylogenetic trees were constructed in MEGA version 7.0, based on the equally weighted neighbor-joining method, and bootstrap values of the phylogenetic tree were calculated with 1000 replicate analyses.

### 2.6. Gene Structure and Conserved Motifs of ACS and ACO Genes

To determine the gene intron/exon structure, *ACS* and *ACO* gene information was implemented from the genome annotations using the online program Gene Structure Display Server 2.0 (GSDS 2.0, http://gsds.gao-lab.org/, accessed on 15 July 2026) [25]. The MEME online program (http://meme.nbcr.net/meme/intro.html, accessed on 15 July 2026) was utilized to predict and characterize conserved motifs within the encoded protein sequences [26]. Gene structures and conserved motifs patterns were visualized with TBtools (version 2.460) [27].

### 2.7. Chromosomal Localization and GENE Duplication

All *ACS* and *ACO* genes were mapped to the pear chromosomes based on the physical location information from the ‘Kuerle Xiangli’ genome (*Pyrus sinkiangensis*) and visualized using TBtools. *ACS* and *ACO* genes expansion models were investigated, including whole genome (WGD) and segmental, tandem, proximal, and dispersed duplications. The Multiple Collinearity Scan toolkit X (MCScanX) was used to analyze the gene duplication events, with the default parameters. To display the synteny analysis of the orthologous *ACS* and *ACO* genes of pear, Arabidopsis, apple, peach, grape, and strawberry, syntenic analysis maps were constructed using the Dual Synteny Plotter in TBtools software (version 2.460). Non-synonymous (Ka) and synonymous (Ks) substitutions of each duplicated *ACS* and *ACO* gene were calculated using TBtools.

### 2.8. RNA Extraction and RT-qPCR Analysis

The total RNA of pear flesh was extracted at different stages according to the instruction of the FastPure Plant Total RNA Isolation Kit (RC401-01, Vazyme, Nanjing, China), following the manufacturer’s instructions. RNA concentration and purity were assessed using a NanoDrop 2000 spectrophotometer (Thermo Fisher Scientific, Waltham, MA, USA), with A260/A280 ratios of 1.8–2.1 and A260/A230 ratios > 2.0 considered acceptable. *RNA* integrity was verified by 1% agarose gel electrophoresis. Reverse transcription was conducted with 1 µg RNA by TransScript^®^ One-Step gDNA Removal and cDNA Synthesis SuperMix (TRANSGEN, Beijing, China). Gene-specific primers were designed with Primer Premier 5.0, and quantitative real-time PCR (qRT-PCR) was carried out with a 20 μL system using SYBR Green Real-Time PCR Master Mix (Takara, Dalian, China) in a LightCycler 480^®^ II/96 Thermal Cycler (Roche Diagnostics, Rotkreuz, Switzerland). The thermal cycling conditions were 95 °C for 3 min, followed by 40 cycles of 95 °C for 10 s and 60 °C for 30 s. Relative expression levels were calculated using the 2^−ΔΔCt^ method [28]. The expression levels of target genes were normalized to the reference gene *Pbr041114.1* in quantitative real-time PCR experiments, as previously reported [29]. Three biological replicates were performed for each analysis. All primer sequences are listed in Table S1.

### 2.9. Data Analyses

All experimental data were initially compiled using Microsoft Excel 2021, and statistical analyses were performed using GraphPad Prism 10.1.2 (GraphPad Software, San Diego, CA, USA). Each treatment had three biological replicates. Data were analyzed as mean ± standard deviation (SD) from at least three biological replicates using SPSS 24.0. For each biological replicate, fruits were independently measured and averaged as one biological replicate. One-way analysis of variance (ANOVA) was applied to determine statistical differences among treatments, followed by Tukey’s honestly significant difference test for multiple comparisons at a significance level of *p* < 0.05, and significant difference (*p*-value < 0.05) was represented by different letters (a, b, c, d, e).

## 3. Results

### 3.1. Ethylene Biosynthesis During Fruit Development in ‘Kuerle Xiangli’

**Figure 1:**
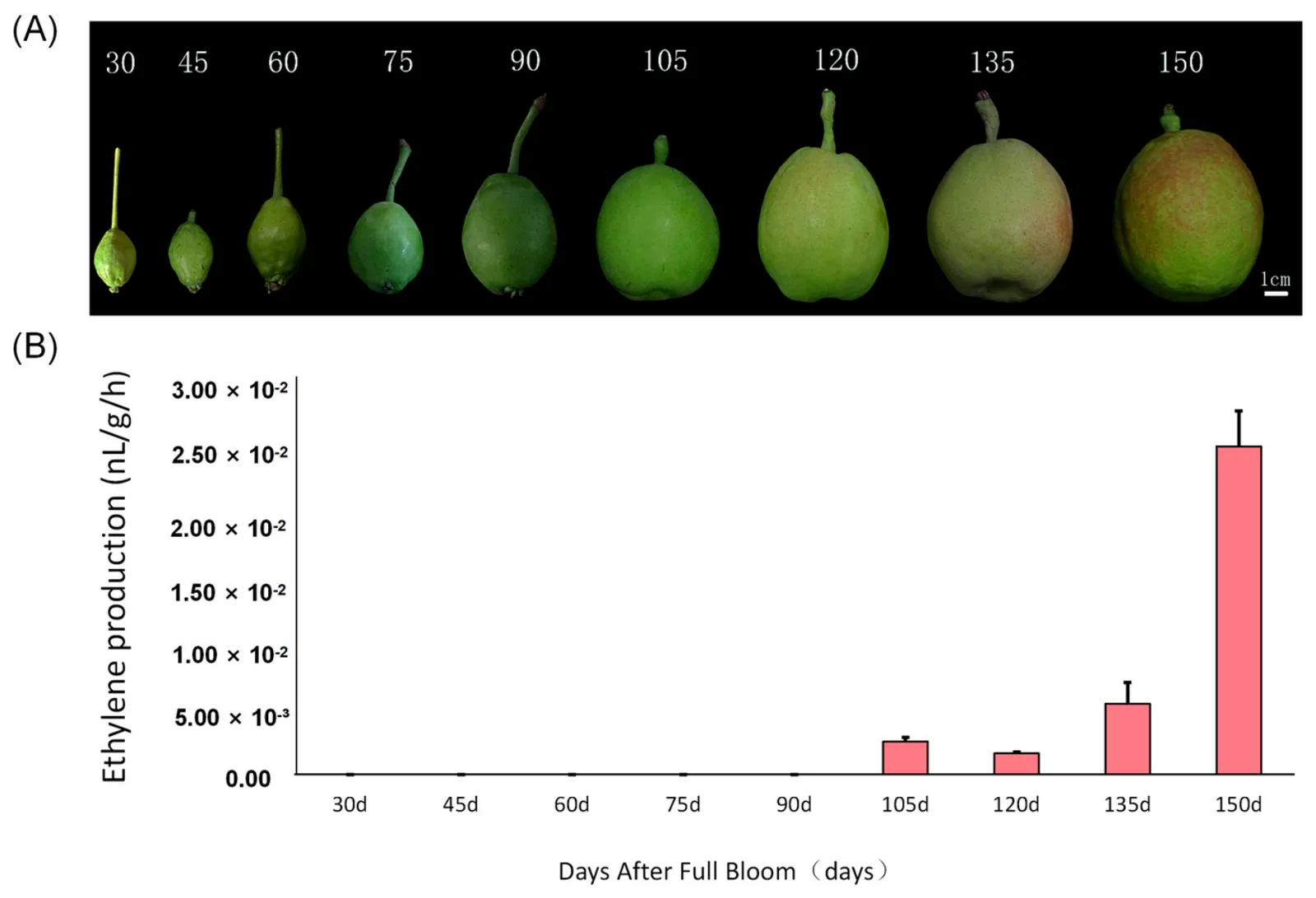
Phenotypic characterization and ethylene production during development in ‘Kuerle Xiangli’. (**A**) Morphological changes of fruit at nine developmental stages from 30 to 150 days after full bloom (DAFB). Scale bar = 1 cm. (**B**) Ethylene production was measured in the fruit at 30, 45, 60, 75, 90, 105, 120, 135 and 150 DAFB and first detected at 105 DAFB. Means and standard errors were calculated using ANOVA.

To clarify the dynamic phenotypic changes and ethylene production during fruit development and ripening, the morphological appearance and ethylene production was investigated in ‘Kuerle Xiangli’ fruit (*Pyrus sinkiangensis*) at nine stages, ranging from fruitlet (30 days after full bloom, DAFB) to ripening (150 DAFB) (Figure 1A). Extremely low basal ethylene levels were detected from 30 to 90 DAFB, with nearly undetectable ethylene emission at these early growth stages. Ethylene synthesis was first recorded at 105 DAFB and output increased at 135 DAFB, and an abrupt dramatic surge of ethylene production occurred at 150 DAFB, reaching the peak value of the entire developmental series (Figure 1B). These results showed ethylene was detected and began to exert its role in the late stage of fruit development and ripening, and also demonstrated that ‘Kuerle Xiangli’ exhibits typical climacteric ripening characteristics; ethylene release was notably higher in ripening fruits compared with developing fruits, remains suppressed during fruit growth and expansion, and a massive ethylene burst specifically occurs at the late ripening stage.

### 3.2. Changes of Fruit Firmness and Soluble Solids Content at Different Stages in ‘Kuerle Xiangli’

**Figure 2:**
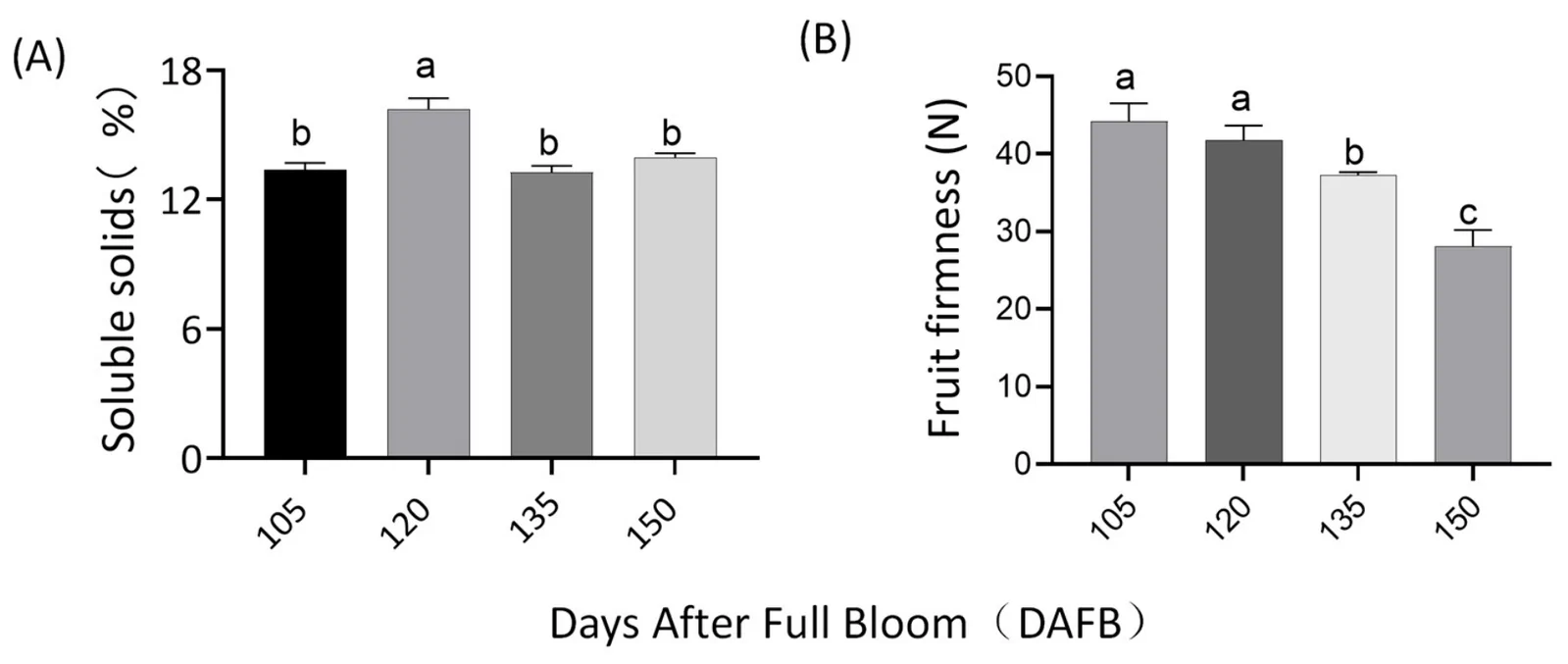
Changes in soluble solids content and fruit firmness during late fruit development and ripening. (**A**) Soluble solids content peaked at 120 DAFB and subsequently declined, with 120 DAFB being significantly higher than all other stages. (**B**) Fruit firmness was tested at 105, 120, 135 and 150 DAFB; this value decreased between 135 and 150 DAFB. Means and standard errors were calculated using ANOVA. Different lowercase letters denote significant differences (*p* < 0.05).

To assess fruit quality attributes in the late developmental and ripening stages, when ethylene starts to be released in large quantities, soluble solids content (SSC) and flesh firmness were measured at 105, 120, 135, and 150 days after full bloom (DAFB). SSC increased significantly from 105 to 120 DAFB, reaching a peak of 16.2% at 120 DAFB, after which it declined to approximately 13.2% at 135 DAFB and remained at 14% at 150 DAFB (Figure 2A). The SSC at 120 DAFB was significantly higher than at all other time points (*p* < 0.05). Fruit firmness remained stable at approximately 8.5–9.0 kg/cm^2^ during the 105–120 DAFB, with no significant difference between these two stages (Figure 2B). However, it decreased significantly to 7.6 kg/cm^2^ at 135 DAFB (*p* < 0.05) and further to 5.7 kg/cm^2^ at 150 DAFB (*p* < 0.05), suggesting fruit firmness decreased significantly at 120 DAFB and maintained this trend until fruit ripening. These results reveal a temporal decoupling between SSC accumulation and textural changes during pear fruit ripening; SSC peaked at 120 DAFB and subsequently declined, whereas firmness was maintained at high levels through 120 DAFB before undergoing a sharp decline from 135 DAFB onward, reflecting the onset of fruit softening during the final ripening stages.

### 3.3. Identification and Analysis of PsinACS Genes in ‘Kuerle Xiangli’

To elucidate the roles of ethylene biosynthesis-related genes during fruit development and ripening, the members of the *ACS* families were identified, which encode the key enzyme ACC synthase in the ethylene biosynthetic pathway. A total of 12 *ACS* genes (named as *PsinACS1*–*PsinACS12*) were isolated from the ‘Kuerle Xiangli’ genome (*Pyrus sinkiangensis*). The coding sequences (CDS) and protein sequences of these genes are provided in Table S2 and their basic information, including Gene ID, genomic start/stop positions, CDS length, protein length, isoelectric point (pI), molecular weight (MW), and gene family classification, are summarized in Table 1. Among the identified *PsinACS* genes, protein length ranged from 159 amino acid (aa) (*PsinACS2*) to 522 aa (*PsinACS9*). pI ranged from 5.36 (*PsinACS4*) to 7.92 (*PsinACS1*). Molecular weight ranged from 17.67 kDa (*PsinACS2*) to 59.03 kDa (*PsinACS9*). Domain analysis against the Pfam database showed that all *PsinACS* proteins contained the aminotransferase class I and II domain (PF00155), with complete domains identified in 11 members (approximately amino acids 39–429). *PsinACS2* contained only a partial PF00155 domain (amino acids 47–158), suggesting that it may be a truncated or non-functional member (Table S5).

**Figure 3:**
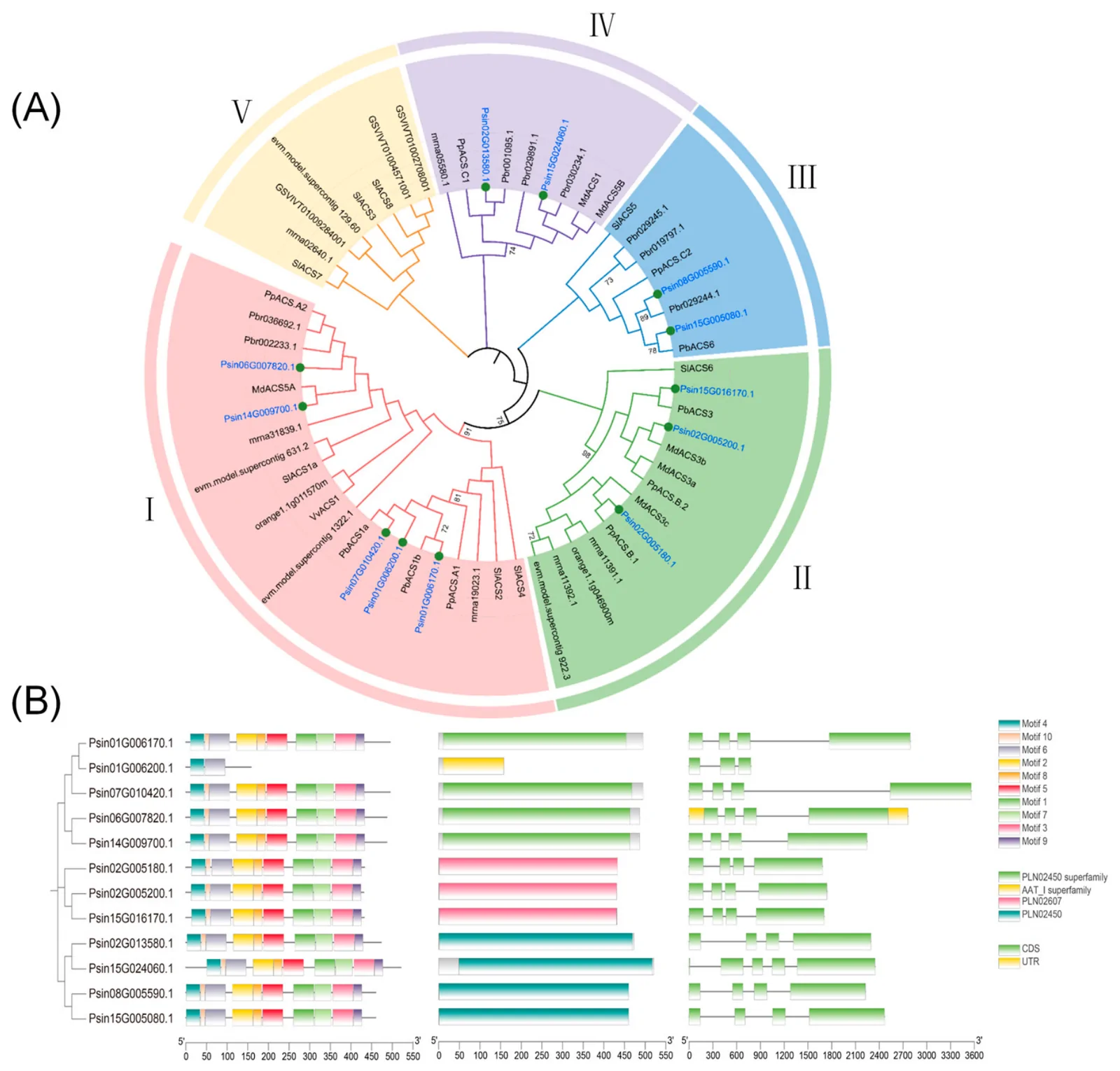
Phylogenetic analysis and structural characterization of the *ACS* gene family in ‘Kuerle Xiangli’. (**A**) A phylogenetic tree of *ACS* proteins isolated from pear (*Pyrus sinkiangensis*, Psin), *P. bretschneideri* (Pb), peach (*Prunus persica*, Pp), apple (*Malus × domestica*, Md), strawberry (*Fragaria × ananassa*, Fa), papaya (*Carica papaya*, Cp), tomato (*Solanum lycopersicum*, Sl), orange (*Citrus sinensis*, Cs), and grape (*Vitis vinifera*, Vv). The tree was generated using MEGA 7.0 with the neighbor joining method. The proteins were clustered into five different groups and marked. (**B**) Phylogenetic relationships, motif pattern, conserved domains, and exon/intron structure of *PsinACS* genes (left to right) from basic information of the *PsinACS* in ‘Kuerle Xiangli’. The motifs, numbers 1–10, are displayed in different colored boxes. The length of protein can be estimated using the scale at the bottom. CDS is highlighted by green boxes and UTR is highlighted by yellow boxes.

To examine evolutionary relationship among the pear and other fruit species (including grape, peach, apple, tomato, papaya, orange, and strawberry), the *ACS* genes were used to construct a phylogenetic tree (neighbor joining method). A total of 64 *ACS* proteins were clearly divided into five groups (Groups I–V) (Figure 3A). Group I was the largest clade, comprising *PsinACS1*, *PsinACS2*, *PsinACS3*, *PsinACS11*, *PsinACS12*, and their homologs from other species. Group II contained *PsinACS4*, *PsinACS5* and *PsinACS6*. Group III comprised *PsinACS7* and *PsinACS8* together with homologs from other species, and Group IV included *PsinACS9* and *PsinACS10*. High bootstrap support values across the tree indicated strong evolutionary conservation between PsinACS proteins and their orthologs. Notably, *PsinACS1*, *PsinACS2* and *PsinACS3* (Group I) clustered with *PbACS1a*, *PbACS1b*, *PpACS.A1*, *SlACS2*, and *SlACS4*, suggesting conserved functions in ethylene biosynthesis. To further assess the evolutionary conservation of ethylene biosynthetic genes across pear species, we identified the closest *P. ussuriensis* ortholog for each *P. sinkiangensis ACS* gene by BLASTP analysis (Table 1). Most *PsinACS* genes possessed a clear ortholog in *P. ussuriensis* (e.g., *PsinACS3*–*PuACS3b*, *PsinACS7*–*PuACS8*, *PsinACS8*–*PuACS9*, and *PsinACS11/PsinACS12*–*PuACS3a*), whereas *PsinACS1* and *PsinACS2* both mapped to *PuACS4*, and *PsinACS9* and *PsinACS10* both to *PuACS1*, reflecting recent tandem/segmental duplication in *P. sinkiangensis*.

To further characterize the conserved motifs of the *PsinACS* proteins, we performed motif analysis using the MEME suite and visualized the results with TBtools. As shown in Figure 3B, a total of 10 conserved motifs were identified, although their distribution varied among *ACS* members. Specifically, motifs 1–10 (arranged as motifs 4, 10, 6, 2, 8, 5, 1, 7, 3, and 9) were detected in nearly all *PsinACS* proteins, with the exception of *PsinACS2*, which lacked several motifs. These conserved motifs likely constitute the characteristic architecture of the *PsinACS* family. The distinct distribution patterns of conserved motifs clearly separated the three phylogenetic subgroups, suggesting functional conservation within each clade and functional divergence between clades. Moreover, gene structure analysis further revealed that nearly all *PsinACS* genes possessed long contiguous CDS regions without discernible UTR segments, except for two genes that harbored short UTR fragments (highlighted in yellow) at their 5′ termini. The consistent gene structure features observed within each monophyletic group corroborated the reliability of the phylogenetic classification.

### 3.4. Identification and Analysis of PsinACO Genes in ‘Kuerle Xiangli’

The members of the *ACO* families were identified, which encode ACC oxidase (ACO), a core catalytic enzyme responsible for the last step of ethylene biosynthesis. A total of five *ACO* genes (named *PsinACO1*–*PsinACO5*) were isolated. The CDS and protein sequences of these genes are provided in Table S3 and their basic information is summarized in Table 2. As for *ACO* genes, their length ranged from 315 (*PsinACO1*, *PsinACO2*) to 323 (*PsinACO3*–*PsinACO5*). The pI of the proteins ranged from 5.05 (*PsinACO3*) to 5.71 (*PsinACO4*). The molecular weight ranged from 35.37 kDa (*PsinACO2*) to 36.54 kDa (*PsinACO4*, *PsinACO5*). All members contain two typical conserved domains: the DIOX_N domain (PF14226) at the N-terminus (approximately amino acids 5–98) and the 2OG-Fe(II) oxygenase superfamily domain (PF03171) at the C-terminus (approximately amino acids 159–253). Both domains are intact in all five *ACO* members, indicating that they possess the typical structural features of functional *ACO* proteins. The highly conserved positions of the domains among members suggest that the *ACO* family is structurally conserved in ‘Kuerle Xiangli’.

**Figure 4:**
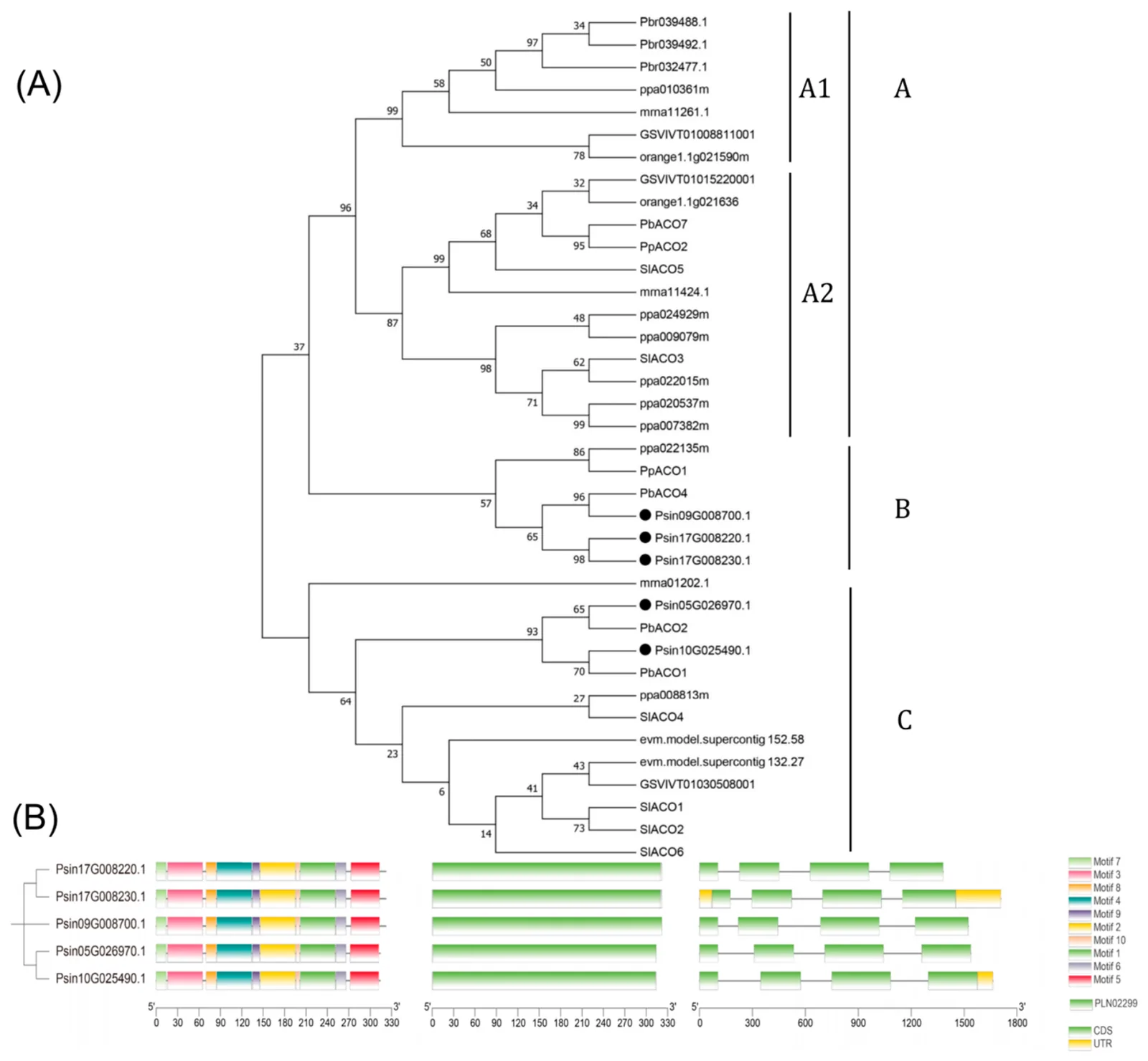
Phylogenetic analysis and structural characterization of the *ACO* protein family in *Pyrus sinkiangensis*. (**A**) A phylogenetic tree of *ACO* proteins isolated from pear (*Pyrus sinkiangensis*, Psin), *P. bretschneideri* (Pb), peach (*Prunus persica*, Pp), apple (*Malus × domestica*, Md), strawberry (*Fragaria × ananassa*, Fa), papaya (*Carica papaya*, Cp), tomato (*Solanum lycopersicum*, Sl), orange (*Citrus sinensis*, Cs), and grape (*Vitis vinifera*, Vv). The tree was generated using MEGA 7.0 with the neighbor joining method. The proteins were clustered into five different groups and marked. (**B**) Phylogenetic relationships, motif pattern, gene structure of five *PsinACO* genes. The motifs are displayed in different colored boxes. The length of protein can be estimated using the scale at the bottom. CDS is highlighted by green boxes and UTR is highlighted by yellow boxes.

Phylogenetic analysis showed that the 38 *ACO* genes were classified into three major groups (Groups A–C) (Figure 4A). Group A were the biggest clade and further divided into two subclusters (A1 and A2). On the contrary, Group B was the smallest clade with six members. Among the five *PsinACO* genes, *PsinACO1* and *PsinACO2* were classified in Group C, whereas *PsinACO3*, *PsinACO4*, and *PsinACO5* were classified in Group B. The result also showed *PsinACO1* and *PsinACO2* (Group C) grouped closely with *PbACO1*, *PbACO2* and *SlACO1*, suggesting strong evolutionary conservation between *PsinACO* and their orthologs and sharing similar functions in pear fruit ripening. Likewise, BLASTP analysis was performed to identify the closest ortholog for each *PsinACO* gene (Table 2). Both *PsinACO1* and *PsinACO2* are orthologous to *PuACO2*, whereas *PsinACO3*–*PsinACO5* correspond to *PuACO3*. These orthologous relationships demonstrate that the *ACO* gene family is largely conserved between the two pear species, and these genes may share similar biological functions. To further examine the structural features underlying the phylogenetic groupings, conserved motif analysis and gene structure characterization were performed for the *PsinACO* genes (Figure 4B). A total of 10 conserved motifs were identified, and their distribution patterns were largely consistent among members within the same clade. No unique clade-specific motifs were exclusively detected in any group, implying functional conservation within each clade. *PsinACO* exhibited uniform length complete CDS regions, reflecting clade-specific structural divergence.

### 3.5. Chromosomal Distribution and Duplication of PsinACS and PsinACO Genes

**Figure 5:**
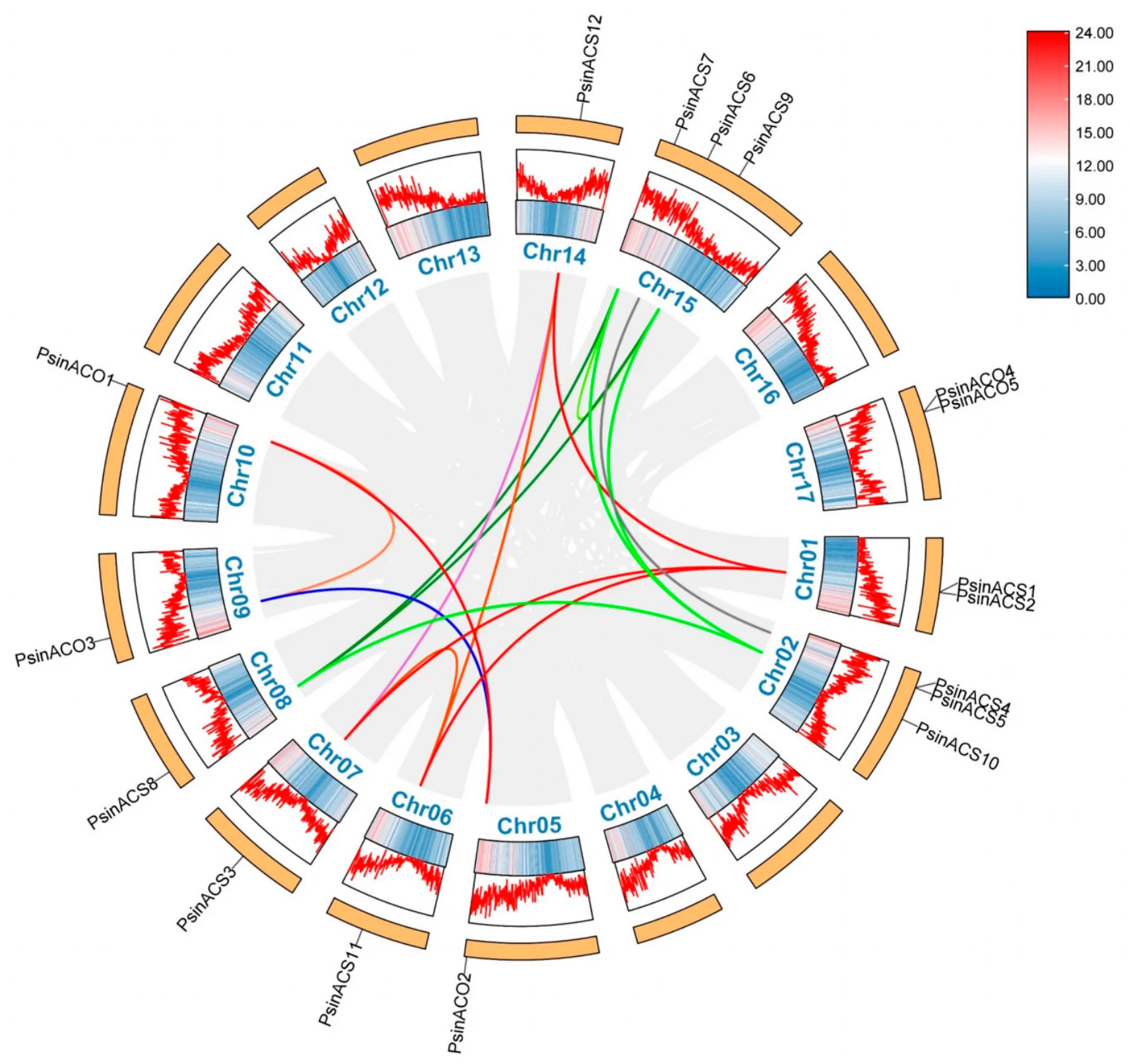
Covariance analysis of pear *PsinACO* and *PsinACS*. The colored lines in the center indicate the syntenic relationships among *PsinACS* and *PsinACO* genes, and different colored lines represent different types of gene duplication events (e.g., red, green, blue, and purple lines represent different types of collinearity).

The *PsinACS* and *PsinACO* genes exhibited an uneven distribution across the seventeen chromosomes of the *Pyrus sinkiangensis* genome (Figure 5). Notably, chromosomes 2 and 15 harbored the largest number of these genes: Chr02 contained *PsinACS4*, *PsinACS5* and *PsinACS10,* while Chr15 carried *PsinACS6*, *PsinACS7* and *PsinACS9*, suggesting these two chromosomes may serve as potential gene-clustering hotspots. Chromosome 1 and chromosome 17 each possessed two genes, with *PsinACS1* and *PsinACS2* located on Chr01, and *PsinACO4* and *PsinACO5* on Chr17. By contrast, no *PsinACS* or *PsinACO* gene was distributed on chromosome 03, 04, 11, 12, 13 or 16. Several other chromosomes (Chr05, Chr06, Chr07, Chr08, Chr09 and Chr10) only harbored a single gene, indicating preferential gene distribution on other chromosomes.

The mode of duplication for the *PsinACS* and *PsinACO* genes was analyzed using MCScanX. As a result, members of the *ACS* and *ACO* gene families were systematically classified into three distinct duplication modes, including whole-genome duplication (WGD)/segmental duplication, proximal duplication, and tandem duplication (Table 3). Most ACS and ACO paralogs arose from ancient WGD or large-scale segmental duplication events, whereas only *PsinACS2* and *PsinACS5* were classified under proximal duplication, while *PsinACO5* exhibited tandem duplication. Collinear homologous gene pairs were identified for several genes, including *PsinACS1* (matched to *Psin06G007820.1*, *Psin07G010420.1*, and *Psin14G009700.1*), *PsinACS3* (matched to *Psin14G009700.1*), *PsinACS4* (matched to *Psin15G016170.1*), *PsinACS7* (matched to *Psin15G024060.1*), *PsinACS8* (matched to *Psin15G005080.1* and *Psin15G024060.1*), *PsinACS10* (matched to *Psin15G005080.1*, *Psin08G005590.1*, and *Psin15G024060.1*), *PsinACS11* (matched to *Psin07G010420.1* and *Psin14G009700.1*), *PsinACO1* (matched to *Psin17G008220.1*), *PsinACO2* (matched to *Psin09G008700.1*, *Psin10G025490.1*, and *Psin17G008220.1*), and *PsinACO3* (matched to *Psin10G025490.1* and *Psin17G008220.1*). No collinear homologs were detected for *PsinACS2*, *PsinACS5*, *PsinACS6*, *PsinACS9*, *PsinACS12*, *PsinACO4*, or *PsinACO5*, suggesting lineage-specific retention or divergence following duplication. These findings highlight the prevalence of WGD/segmental events in shaping the pear ethylene biosynthesis gene family and underscore the role of conserved synteny in maintaining functional redundancy or neofunctionalization.

### 3.6. Synteny Analysis of PsinACS and PsinACO Genes

**Figure 6:**
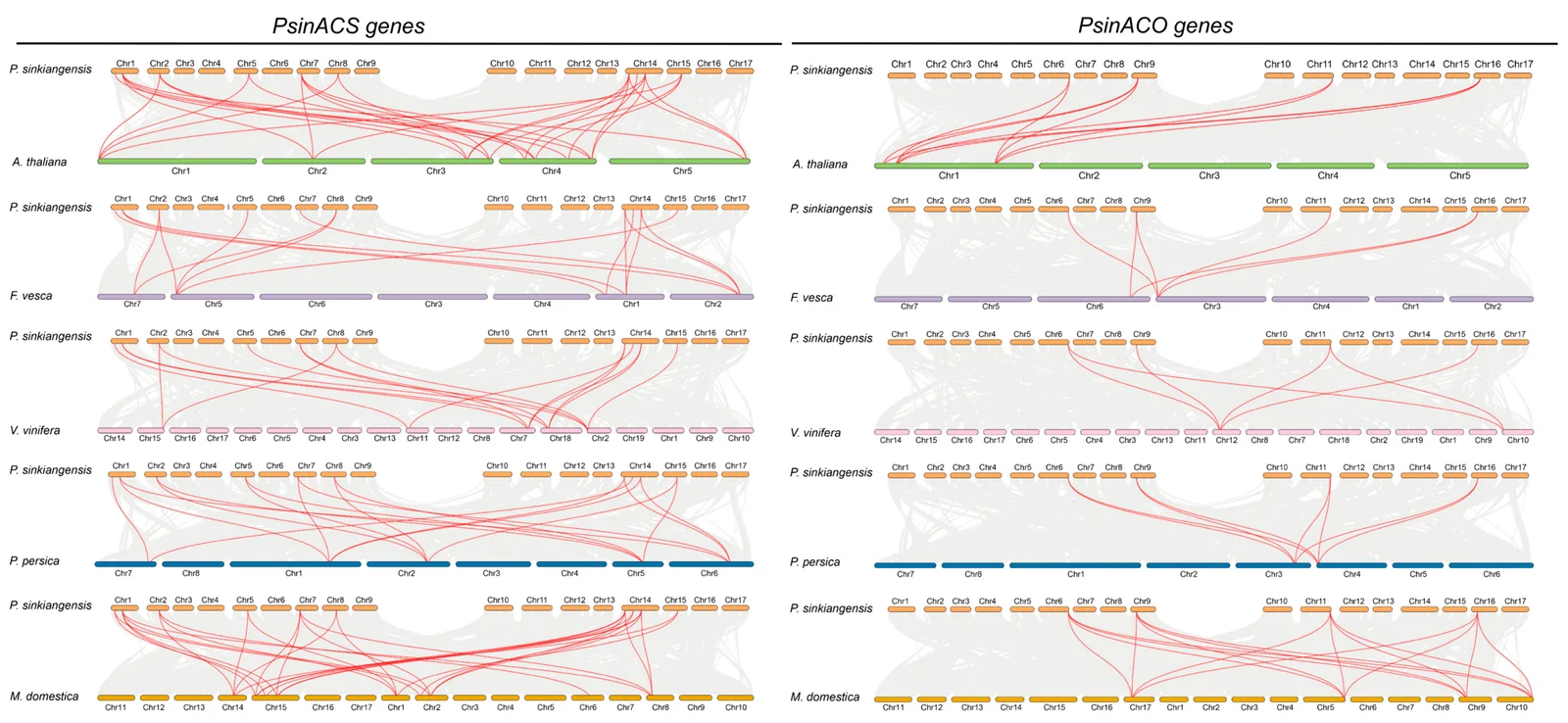
Synteny analysis of *PsinACS* and *PsinACO* genes between pear and five representative plant species. Gray lines in the background indicate the collinear blocks within pear and other plant genomes, while the red lines highlight the syntenic *PsinACS* and *PsinACO* gene pairs. The specie names with ‘*A. thaliana*’, ‘*F. vesca*’, ‘*V. vinifera*’, ‘*P. persica*’, and ‘*M. domestica*’ indicated Pyrus *sinkiangensis*, *Arabidopsis thaliana*, *Fragaria vecsa*, *Vitis vinifera*, *Prunus persica*, and *Malus domestica*.

To further explore the phylogenetic evolutionary mechanisms of *PsinACS* and *PsinACO* genes, comparative syntenic maps were constructed with five representative species: Arabidopsis, strawberry, grape, peach, and apple (Figure 6). The numbers of orthologous pairs between the *ACS* genes and those of the other five species were: Arabidopsis 28, strawberry 15, apple 29, peach 18, and grape 16 (Table S4). Significantly, the results showed that some collinear *ACS* gene pairs shared by pear and apple were anchored to the highly conserved syntenic blocks (Figure 6), which spanned more than 20 genes. In contrast, pear and strawberry had only 15 orthologous *ACS* gene pairs. Interestingly, Only the collinear pairs corresponding to Psin*ACS2* were displayed between pear and apple; for pear versus other species, no such collinear blocks were detected. The numbers of orthologous pairs between the *ACO* genes and those of the other five species were: Arabidopsis 10, strawberry 6, apple 16, peach 8, and grape 6 (Table S4). However, no orthologs of *PsinACO5* could be detected in strawberry, peach, apple, grape or Arabidopsis.

To better understand the evolutionary constraints that have acted on the *ACS* and *ACO* families, the classical measure nonsynonymous (Ka)/synonymous substitution (Ks) ratios of the *ACS* and *ACO* gene pairs were calculated (Table S4). The ratio is associated with the magnitude and direction of selective constraints (Ka/Ks >1, =1, and <1) and indicated neutral evolution, purifying selection, and positive diversifying selection, respectively. The results showed all *PsinACS* genes, and those of the other five species, had Ka/Ks < 1, suggesting that the *ACS* and *ACO* families might have experienced strong purifying selective pressure during evolution.

### 3.7. Expression Patterns of PsinACS and PsinACO Genes in Different Fruit Development Stages

**Figure 7:**
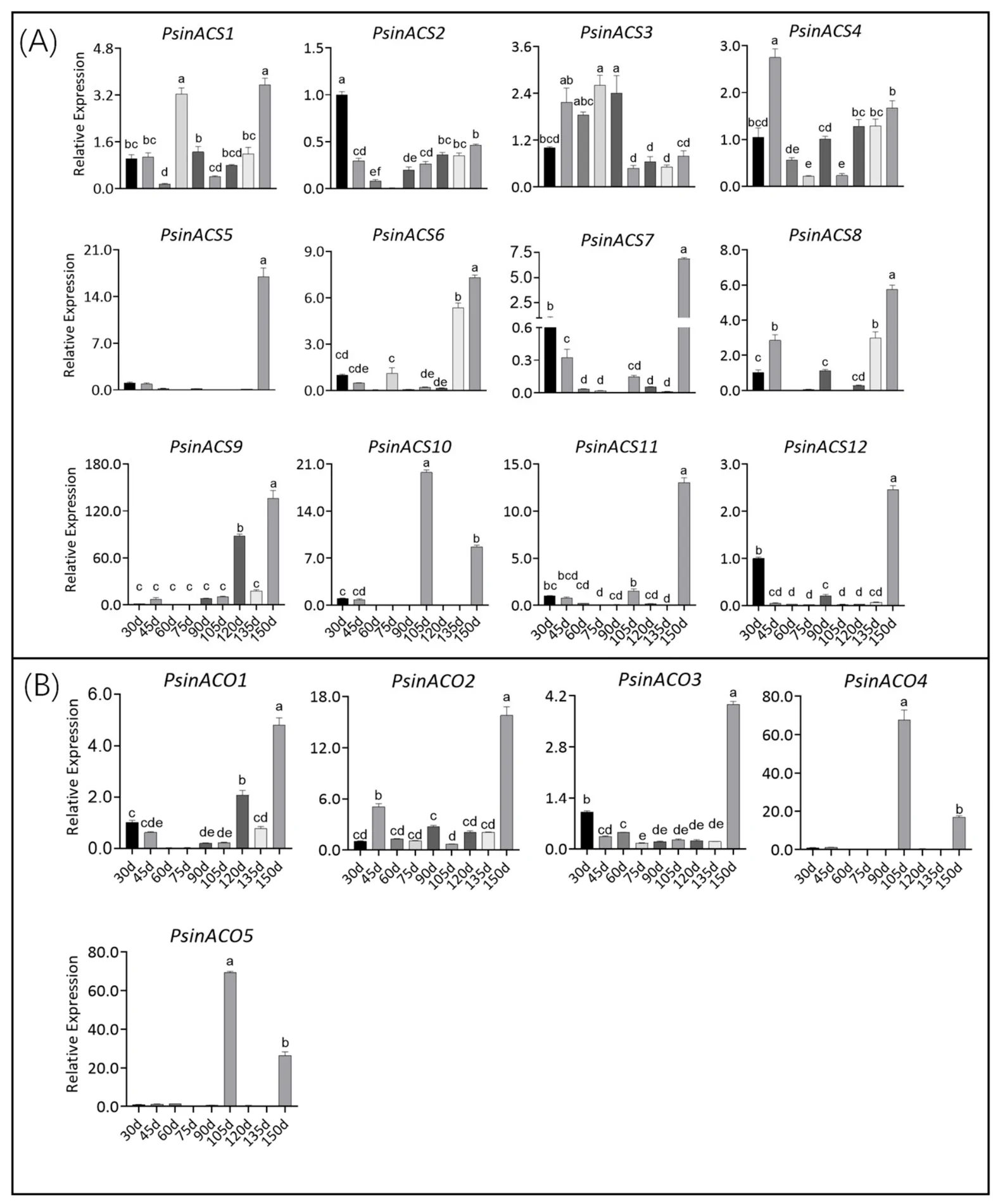
Expression patterns of ethylene biosynthesis-related *PsinACS* and *PsinACO* genes during fruit development. (**A**) *PsinACS* and (**B**) *PsinACO* expression patterns were tested. The fruit for qRT-PCR analysis was collected at 30, 45, 60, 75, 90, 105,120,135 and 150 days after full blooming (DAFB). Means and standard errors were calculated using ANOVA. Different lowercase letters denote significant differences (*p* < 0.05).

To identify the crucial genes that are involved in ethylene biosynthesis, the expression patterns of *PsinACS and PsinACO* genes were examined during different developmental stages of pear fruit, and analyzed by RT-qPCR analysis (Figure 7). As shown in Figure 7A, the expression patterns of the *PsinACS* genes were investigated in nine stages and varied greatly across them, which indicated that they may have different functions in the diverse growth stages of the pear fruit. Among these genes, expressions of *PsinACS2*, *PsinACS3*, and *PsinACS4* were high in the fruitlet stage but decreased gradually in the subsequent developmental stages. *PsinACS10* displayed a relatively strong expression at 105d, indicating that it participated in the fruit enlargement (Figure 7A). Moreover, the expression of *PsinACS1*, *PsinACS5*, *PsinACS6*, *PsinACS7*, *PsinACS8*, *PsinACS9*, *PsinACS11* and *PsinACS12* were more highly expressed at 150d compared with other stages, implying their roles in later fruit development and ripening. Moreover, *PsinACS1* were more highly expressed in pear fruit compared with other members within their respective gene families, suggesting this gene plays a crucial role in the process of ethylene biosynthesis. The qRT-PCR analysis also showed the expression levels of *PsinACO* genes (Figure 7B). *PsinACO1* and *PsinACO2* were markedly upregulated at the ripening stage, suggesting they are the primary functional genes driving ethylene synthesis in ripening fruit. *PsinACO4* and *PsinACO5* were observed with the highest expression level during the middle stages of fruit enlargement, implying that they might play a role during this stage.

### 3.8. Correlation Analysis Among Ethylene Production, Firmness, Soluble Solids Content (SST), and the Expression of ACS and ACO Genes

**Figure 8:**
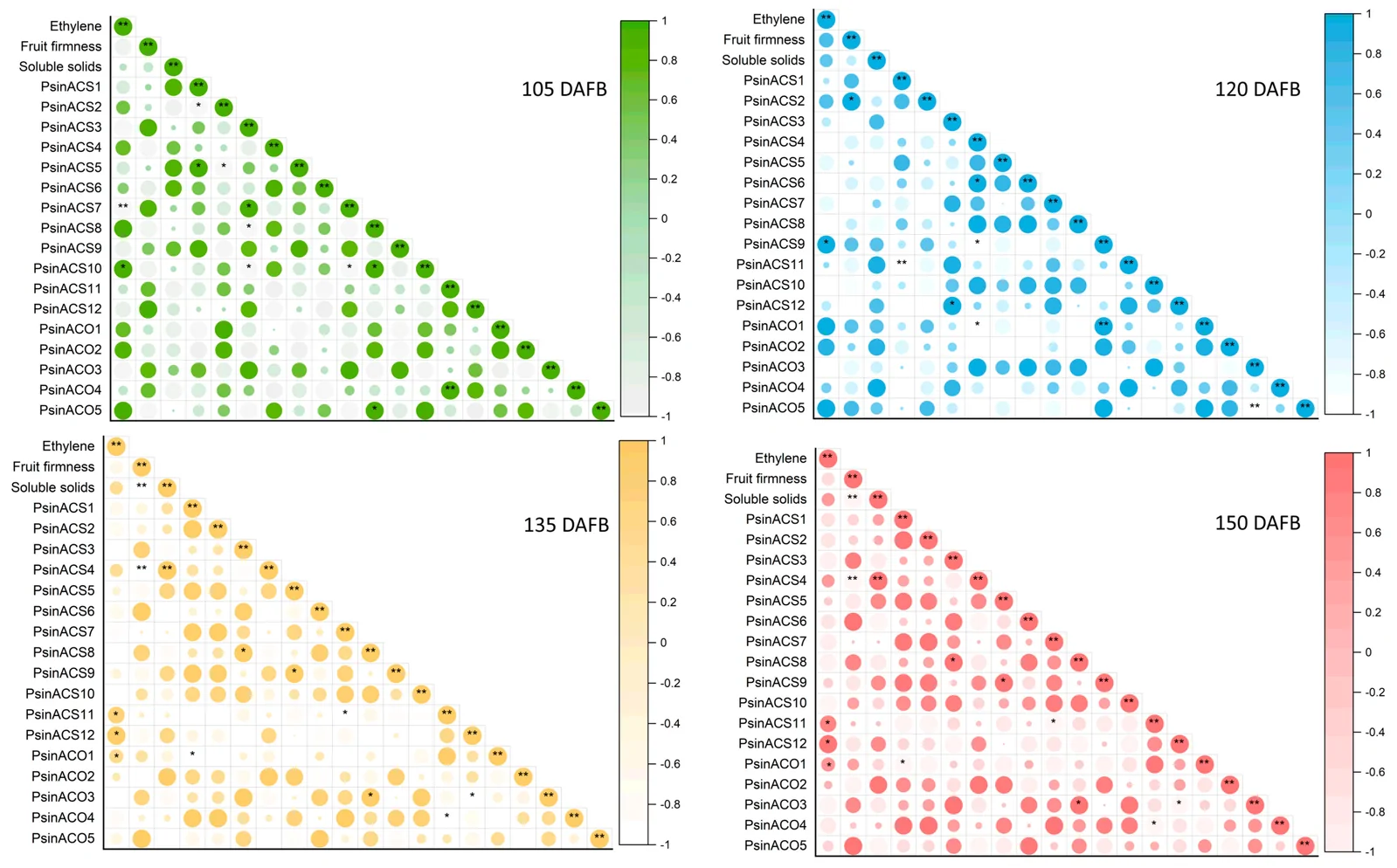
Correlation analysis among ethylene production, fruit firmness, soluble solids content (SSC), and the expression of *PsinACS* and *PsinACO* genes during different stages. Pearson correlation coefficients were calculated between each pair of physiological traits and ethylene biosynthetic genes at 105, 120, 135, and 150 DAFB, and are presented as colored circles in a lower triangular matrix. The color scale indicates the value of the correlation coefficient (*r*), ranging from −1 to 1 as shown on the right of each panel. Significance of the correlations is indicated by asterisks: * *p* < 0.05 and ** *p* < 0.01.

To further clarify the relationships among ethylene production, fruit firmness, soluble solids content (SSC), and the expression of ethylene biosynthetic genes, Pearson correlation analysis was performed at four developmental stages (105, 120, 135, and 150 DAFB; Figure 8). At 105 DAFB, the results revealed a significant positive correlation between ethylene production and the expression of *PsinACS10* (*p* < 0.05), and a significant negative correlation between ethylene production and the expression of *PsinACS7* (*p* < 0.01). No significant correlation was observed between ethylene production, fruit firmness and SSC. These results indicated that the ethylene biosynthetic genes were not yet coordinated with the physiological changes during the early stage of fruit development. At 120 DAFB, ethylene production showed a significant positive correlation with the expression of *PsinACS9*, and *PsinACO1* presented a positive correlation with ethylene and *_PsinACS9.* At 135 and 150 DAFB, a significant negative correlation between fruit firmness and SSC (*p* < 0.01) was presented. Ethylene production showed significant positive correlations with the expression of several *PsinACS* and *PsinACO* genes, such as *PsinACS11, PsinACS12_* and *PsinACO1*, and a negative correlation with fruit firmness, accompanied by a positive correlation with SSC. This pattern suggested that the ethylene climacteric driven by *ACS*/*ACO* upregulation was closely associated with fruit softening and the accumulation of soluble solids. In addition, some members of the *PsinACS* and *PsinACO* gene families were significantly and positively or negatively correlated with one another, indicating a coordinated expression pattern within the ethylene biosynthetic pathway.

## 4. Discussion

### 4.1. ‘Kuerle Xiangli’ Exhibits Typical Climacteric Ripening Behavior

Fruit ripening is a sophisticated and tightly coordinated biological process that involves physiological and metabolic modifications in multiple aspects, including peel color, stone cells, flavor, aroma, texture and plant hormones [30]. For climacteric fruits, ripening is primarily governed by an autocatalytic process involving ethylene [31]. In this study, ethylene production remains suppressed during fruit growth and expansion, followed by a massive ethylene burst specifically at the ripening stage (150 DAFB, Figure 1B); the result demonstrates that ‘Kuerle Xiangli’ exhibits a typical climacteric fruit. A comparable pattern has been documented in other pears, including ‘Nanguo’ (*P. ussuriensis*), in which ethylene remains low until the ripening transition and then rises sharply together with the induction of ripening-associated *ACS*/*ACO* genes [7], and in ‘Bartlett’ (*P. communis*), in which the acquisition of ripening capacity coincides with the establishment of a functional ethylene biosynthetic system near to commercial harvest [32]. It is likewise consistent with the general behavior of tomato, banana and kiwifruit, in which the ripening burst is initiated by the transcriptional activation of specific *ACS* and *ACO* genes [6,9,33]. The detectable ethylene at 105 DAFB suggests that the onset of the ethylene biosynthetic machinery is initiated prior to the climacteric peak, a phenomenon similarly observed where system I ethylene (auto-inhibitory) precedes the system II (auto-catalytic) ethylene production [13].

### 4.2. Temporal Decoupling Between Soluble Solids Accumulation and Firmness Loss

One of the more informative findings of this study is the temporal asynchrony between the two quality changes (Figure 2). SSC rose steeply between 105 and 120 DAFB, peaked at 16.2% at 120 DAFB (significantly higher than at all other stages, *p* < 0.05), and then declined to approximately 13.2% at 135 DAFB before stabilizing at about 14% at 150 DAFB. In contrast, firmness was maintained at 8.5–9.0 kg/cm^2^ through 120 DAFB and only began to fall sharply thereafter (7.6 kg/cm^2^ at 135 DAFB and 5.7 kg/cm^2^ at 150 DAFB). Similar ripening-associated firmness loss has been well documented, where cell wall-modifying enzymes such as polygalacturonase (PG), pectin methylesterase (PME), and expansins are transcriptionally upregulated during softening in tomato [34] and apple [35]. Sugar accumulation reached its maximum roughly 30 days before the major loss of firmness, and the steepest softening occurred during the very interval (135–150 DAFB) in which the ethylene climacteric burst was generated. This decoupling indicates that, in ‘Kuerle Xiangli’, the accumulation of soluble solids and the disassembly of the cell wall are not strictly co-regulated, even though both ultimately contribute to ripening quality.

Two aspects deserve emphasis. First, the decline of SSC after 120 DAFB is unlikely to reflect a genuine loss of sugar; more plausibly, it reflects the combined effect of continued respiratory consumption of soluble sugars during the high-respiration ripening phase and of progressive mobilization/dilution as ripening advances. Second, the lag between maximum SSC and maximum softening is consistent with the view that, in pear, cell wall disassembly is a late ethylene-dependent event [36]. In ‘La France’ and ‘Bartlett’ pear, softening is both initiated and sustained by ethylene perception, and blocking ethylene action with 1-MCP strongly delays firmness loss and the expression of cell wall-modifying genes [32,36]. The temporal pattern observed in this study supports that sugar accumulation primarily reflects early fruit maturation, whereas the final decline in firmness is tightly coupled to the climacteric ethylene burst.

### 4.3. Genome-Wide Identification and Evolutionary Features of PsinACS and PsinACO Gene Families

ACS and ACO act as two rate-limiting enzymes in the ethylene biosynthetic pathway, whose expression levels directly determine the total ethylene production of climacteric fruits. We identified 12 *ACS* and 5 *ACO* genes in the ‘Kuerle Xiangli’ (*P. sinkiangensis*) genome, numbers comparable to those reported in other Rosaceae species. For instance, 12 *ACS* and 11 *ACO* genes were identified in *Pyrus ussuriensis* [7]. In tomato, 9 *LEACS* genes and 4 *LEACO* genes have been identified [6] and 19 *ACS* genes have been reported in apple [37], indicating that these gene families are generally conserved across the plant. Comparison with the *ACS* and *ACO* gene families of *P. ussuriensis* [9] further supports the functional conservation of ethylene biosynthesis across pear species. The ripening-upregulated *PsinACS1*, *PsinACS5*, *PsinACS11* and *PsinACS12* correspond to *PuACS4*, *PuACS2*/*PuACS11* and *PuACS3a*, respectively, whose ripening-associated induction has been described in [7], consistent with a conserved role of these Group I/II members in the climacteric ethylene burst. Likewise, the ripening-induced *PsinACO1* and *PsinACO2* share the ortholog *PuACO2*, whereas the *PsinACO4* and *PsinACO5* ortholog to *PuACO3*, suggesting a broader developmental role for this member. Together, these comparisons indicate that the core ethylene biosynthetic program is largely conserved between *P. sinkiangensis* and *P. ussuriensis,* with individual paralogs exhibiting distinct developmental expression patterns.

Phylogenetic analysis grouped the *PsinACS* proteins into four main clades (Groups I–IV). Group I, the largest clade, contained *PsinACS1*, *PsinACS2*, *PsinACS3*, *PsinACS11*, and *PsinACS12*, along with their orthologs from apple (*MdACS5A*), grape (*VvACS1*), peach (*PpACS.A1*, *PpACS.A2*), and tomato (*SlACS1a*, *SlACS2*, *SlACS4*). Notably, several members of Group I have been functionally characterized as key ripening-associated *ACS* genes across multiple species. For example, *SlACS2* and *SlACS4* are crucial for autocatalytic ethylene production during tomato fruit ripening. *SlACS2*, *SlACS4* and *SlACS1a* exhibit significantly elevated expression levels associated with fruit ripening [38]. Overexpression of transcription factor *SlbHLH22* in tomato promotes ethylene production and activates these ripening-related genes. NOR-like1 controls ethylene biosynthesis by binding to the promoter regions of *SlACS2* and *SlACS4* [39,40]. *PbACS1a/b* are significantly associated with ethylene biosynthesis and fruit ripening in Pyrus *bretschneideri* [22]. The *PsinACO* proteins fall into Groups B and C, with *PsinACO1* and *PsinACO2* clustering with the ripening-related *PbACO1* and *SlACO1* (Group C) [22,41]. The conservation of these phylogenetic relationships strongly suggests that the pear genes clustered in the ripening-associated clades share conserved functions in ethylene biosynthesis. The presence of *PsinACO3*, *PsinACO4* and *PsinACO5* outside of these defined groups may indicate a functionally divergent member, potentially involved in stress responses or other developmental processes.

Gene duplication analysis revealed that most *PsinACS* and *PsinACO* paralogs arose from whole-genome or segmental duplication events, with only a few derived from proximal (*PsinACS2*, *PsinACS5*) or tandem (*PsinACO5*) duplication. Synteny analysis confirmed a strong evolutionary conservation of the *ACS* and *ACO* loci across Rosaceae species, and the Ka/Ks ratios (<1) of all analyzed gene pairs indicated that both gene families have been under strong purifying selection. Together, these findings indicate that the *ACS* and *ACO* families in pear have been functionally constrained during evolution, with whole-genome/segmental duplication as the primary force driving family expansion and purifying selection maintaining functional redundancy among paralogs, a scenario consistent with genome-wide analyses in *P. ussuriensis* and other Rosaceae fruits [7,37,42].

### 4.4. Correlation Analysis Links Ethylene Production with Fruit Quality and ACS/ACO Expression

To move from a descriptive to a more quantitative integration of the physiological, biochemical, and molecular data, we performed Pearson correlation analyses among ethylene production, firmness, SSC, and the expression of all *PsinACS* and *PsinACO* genes at the four ripening-associated stages (105, 120, 135, and 150 DAFB; Figure 8). The results revealed stage-dependent associations. At 105 DAFB, when ethylene had just become detectable, ethylene production was significantly positively correlated with *PsinACS10* expression (*p* < 0.05) and significantly negatively correlated with *PsinACS7* (*p* < 0.01), but no significant association with firmness or SSC was detected, indicating that the ethylene biosynthetic machinery was not yet coordinated with the physiological changes at this early stage. At 120 DAFB, ethylene production was positively correlated with *PsinACS9*, and *PsinACO1* expression was positively correlated with both ethylene and PsinACS9. At the climacteric stages (135 and 150 DAFB), ethylene production became significantly positively correlated with several ripening-associated genes (notably *PsinACS11*, *PsinACS12*, and *PsinACO1*), was negatively correlated with firmness and positively correlated with SSC, and firmness and SSC were negatively correlated with each other (*p* < 0.01). These stage-dependent shifts in the correlation structure are consistent with the physiological observation that the linkage among ethylene, softening, and sugar accumulation is established only as ripening progresses. Additionally, Pearson correlation analysis revealed that the transcript abundance of *PsinACS4* was extremely significantly positively correlated with soluble solids content and extremely significantly negatively correlated with flesh firmness. The gene–gene correlations within the *ACS*/*ACO* families may reflect shared transcriptional regulation by common upstream factors [14,15,16,19] rather than direct interdependence.

It is important to emphasize that correlation analysis establishes statistical associations rather than biological causation. The observed correlations, particularly the positive associations between ethylene and *PsinACS11*, *PsinACS12*, and *PsinACO1* and the negative association between ethylene and firmness, support, but do not by themselves prove, a causal role for these genes in the ethylene climacteric and in softening. A causal interpretation is, however, strongly supported by three independent lines of evidence: (i) the temporal coincidence between the upregulation of these genes and the ethylene burst at 150 DAFB; (ii) the functional validation of their orthologs in other species, where silencing or overexpression of the corresponding *ACS/ACO* genes alters ethylene production and ripening (for example, *SlACS2*/*SlACS4* and *SlACO1* in tomato [39,40,41,43]; *PbACS1a/b* in *P. bretschneideri* [22]); and (iii) the identification of upstream transcriptional regulators, such as *PbrERF114*-*PbrACS3/PbrACO1* and *PuWRKY31–PuACS1a/PuACO1* regulatory modules [16,19]. Taken together, the correlation network among the physiological traits and the ethylene biosynthetic genes progressively strengthened during ripening, supporting the view that the coordinated upregulation of *PsinACS* and *PsinACO* genes underpins the ethylene climacteric, which in turn mediates fruit softening and sugar accumulation.

### 4.5. Promoter Cis-Regulatory Elements Reveal Multi-Hormonal and Stress-Responsive Regulation of ACS and ACO Genes

The 2000 bp upstream promoter regions of the 12 *PsinACS* and 5 *PsinACO* genes were analyzed using the PlantCARE database to identify putative cis-acting regulatory elements (Figure S1). The promoter analysis of *PsinACS* and *PsinACO* genes uncovered a diverse array of cis-regulatory elements, with the most abundant categories being hormone-responsive elements, such as abscisic acid responsiveness, gibberellin responsiveness, auxin responsiveness, salicylic acid responsiveness, and MeJA responsiveness. The enrichment of ABRE (ABA-responsive), CGTCA-motif (MeJA-responsive), and TGA-element (auxin-responsive) elements in *ACS*/*ACO* promoters points to potential cross-talk between ethylene and other hormonal signaling pathways during pear fruit ripening. This is consistent with accumulating evidence that ABA–ethylene synergism [44,45] and JA–ethylene interaction [46] cooperatively regulate climacteric fruit ripening. ERF transcription factor *PbrERF114* directly binds GCC-box elements in the *PbrACS3* and *PbrERF24* promoters, forming a transcriptional cascade that amplifies ethylene synthesis [16]. Our identification of ERF-binding elements in multiple *PsinACS*/*PsinACO* promoters suggests that additional ERF-mediated regulatory modules may exist beyond the ERF pathway.

The presence of stress-responsive elements (LTR, MBS) in several *PsinACS* and *PsinACO* promoters also raises the intriguing possibility that ethylene biosynthesis in pear may be modulated by environmental stresses such as low temperature and drought, as has been documented in other species. For instance, in citrus, *ACO1* expression is activated through chromatin remodeling under polyploidization and salt stress [47], while in banana, *MaACS1* is significantly upregulated under low potassium conditions [48]. These observations collectively underscore the integration of developmental and environmental signals at the promoter level of ethylene biosynthetic genes.

## 5. Conclusions

In this study, we characterized the ethylene biosynthesis dynamics and the *ACS* and *ACO* gene families in ‘Kuerle Xiangli’ (*Pyrus sinkiangensis*) during fruit development. Our results demonstrate that ‘Kuerle Xiangli’ exhibits a typical climacteric ripening pattern, with a pronounced ethylene burst occurring at 150 DAFB. Our results show that this cultivar undergoes typical climacteric ripening, and its ripening process is accompanied by fruit softening and soluble solids accumulation.

We identified 12 *PsinACS* and 5 *PsinACO* genes and revealed evolutionary conservation with other species, with WGD/segmental duplication as the primary driving force for the expansion of these gene families. Expression profiling showed *PsinACS1* and *PsinACO1* are crucial genes in ethylene biosynthesis that are highly expressed at the ethylene climacteric and positively regulate fruit ripening. Collectively, these findings establish a framework for understanding the molecular regulation of ethylene biosynthesis during pear fruit ripening and provide a valuable gene resource for molecular breeding in pear.

## Figures and Tables

**Table 1:** Basic information of the *PsinACS* in ‘Kuerle Xiangli’.

Gene Name	Gene ID	Start	Stop	CDS Length (bp)	Protein Length (aa)	pI	Molecular Weight (kDa)	Gene Family	Orthologs (*P. ussuriensis*)
*PsinACS1*	*Psin01G006170.1*	13804122	13806914	1488	496	7.92	55.52	Group I	*PuACS4*
*PsinACS2*	*Psin01G006200.1*	13857116	13857895	477	159	6.39	17.67	Group I	*PuACS4*
*PsinACS3*	*Psin07G010420.1*	15598219	15601780	1488	496	7.96	55.49	Group I	*PuACS3b*
*PsinACS4*	*Psin02G005180.1*	4190137	4191821	1320	440	5.36	48.63	Group II	*PuACS7*
*PsinACS5*	*Psin02G005200.1*	4209810	4211550	1296	432	5.82	48.17	Group II	*PuACS2*, *PuACS11*
*PsinACS6*	*Psin15G016170.1*	12926876	12928581	1299	433	5.47	48.42	Group II	*PuACS7*
*PsinACS7*	*Psin15G005080.1*	3707636	3710102	1383	461	7.37	52.43	Group III	*PuACS8*
*PsinACS8*	*Psin08G005590.1*	5008619	5010846	1383	461	6.28	52.13	Group III	*PuACS9*
*PsinACS9*	*Psin15G024060.1*	21992876	21995223	1566	522	7.42	59.03	Group IV	*PuACS1*
*PsinACS10*	*Psin02G013580.1*	12822840	12825136	1422	474	7.56	53.19	Group IV	*PuACS1*
*PsinACS11*	*Psin06G007820.1*	16728438	16731201	1464	488	7.29	54.76	Group I	*PuACS3a*
*PsinACS12*	*Psin14G009700.1*	16272904	16275150	1464	488	7.29	54.85	Group I	*PuACS3a*

**Table 2:** Basic information of the *PsinACO* in ‘Kuerle Xiangli’.

Gene Name	Gene ID	Start	Stop	CDS Length (bp)	Protein Length (aa)	pI	Molecular Weight (kDa)	Gene Family	Orthologs (*P. ussuriensis*)
*PsinACO1*	*Psin10G025490.1*	33335094	33336757	945	315	5.05	35.44	Group B	*PuACO2*
*PsinACO2*	*Psin05G026970.1*	33778976	33780514	945	315	5.16	35.37	Group B	*PuACO2*
*PsinACO3*	*Psin09G008700.1*	6636244	6637768	969	323	5.08	36.42	Group B	*PuACO3*
*PsinACO4*	*Psin17G008220.1*	6988396	6989777	969	323	5.71	36.54	Group C	*PuACO3*
*PsinACO5*	*Psin17G008230.1*	7005472	7007180	969	323	5.71	36.54	Group C	*PuACO3*

**Table 3:** The duplication events and collinear homologous genes of *ACS* and *ACO* gene families in ‘Kuerle Xiangli’.

Gene Name	The Duplicate Event	Matched Collinear Homologous Gene ID
*PsinACS1*	WGD or Segmental	*Psin06G007820.1*, *Psin07G010420.1*, *Psin14G009700.1*
*PsinACS2*	Proximal	
*PsinACS3*	WGD or Segmental	*Psin14G009700.1*
*PsinACS4*	WGD or Segmental	*Psin15G016170.1*
*PsinACS5*	Proximal	
*PsinACS6*	WGD or Segmental	
*PsinACS7*	WGD or Segmental	*Psin15G024060.1*
*PsinACS8*	WGD or Segmental	*Psin15G005080.1*, *Psin15G024060.1*
*PsinACS9*	WGD or Segmental	
*PsinACS10*	WGD or Segmental	*Psin15G005080.1*, *Psin08G005590.1*, *Psin15G024060.1*
*PsinACS11*	WGD or Segmental	*Psin07G010420.1*, *Psin14G009700.1*
*PsinACS12*	WGD or Segmental	
*PsinACO1*	WGD or Segmental	*Psin17G008220.1*
*PsinACO2*	WGD or Segmental	*Psin09G008700.1*, *Psin10G025490.1*, *Psin17G008220.1*
*PsinACO3*	WGD or Segmental	*Psin10G025490.1*, *Psin17G008220.1*
*PsinACO4*	WGD or Segmental	
*PsinACO5*	Tandem	

## Data Availability

All data generated and analyzed during this study were included in this published article and its Supporting Information Files (Supplementary Tables S1–S5). Moreover, all other data are available from the corresponding author upon reasonable request.

## Supplementary Materials

The following supporting information can be downloaded at: https://www.mdpi.com/article/10.3390/biology15191769/s1. Figure S1: Cis-regulatory element analysis of the promoters of *PsinACS* and *PsinACO* genes in Pyrus sinkiangensis; Table S1: primer sequences; Table S2: Information on CDS and protein sequences of *PsinACS* genes; Table S3: Information on CDS and protein sequences of *PsinACO* genes; Table S4: Nonsynonymous (Ka)/synonymous (Ks) substitution ratios of *ACS* and *ACO* gene pairs. Table S5: The identified Pfam domains of *PsinACS* and *PsinACO*.

## Abbreviations

The following abbreviations are used in this manuscript:

SSC	Soluble solids content
ACC	1-aminocyclopropane-1-carboxylic acid
ACS	ACC synthase
ACO	ACC oxidase
DAFB	Days after full bloom
CDS	Coding sequences
MW	Molecular weight
