# Supplementary material for: Ethylene Biosynthesis Dynamics and Genome-Wide Characterization of the ACS and ACO Gene Families During Fruit Development in ‘Kuerle Xiangli’ (Pyrus sinkiangensis Yu)
Source: Biology (Basel). 2026 Oct 4;15(19):1769. doi: 10.3390/biology15191769 (PMC13664455; doi:10.3390/biology15191769)
Supplement: Supplementary file 1 [file biology-15-01769-s001.zip › Figure S1 Cis-regulatory element analysis of the promoters of PsinACS and PsinACO genes in Pyrus sinkiangensis..pdf]

Ethylene Biosynthesis Dynamics and Genome-wide Characterization of the ACS and ACO Gene Families During Fruit Development in 'Kuerle Xiangli' (*Pyrus sinkiangensis* Yu)

Tingting Fan [1,2,3], Deen Zhang [1,2,3], Wenjie Li [1], Zhihua Guo [1,2,3,*] and Xiaoyan Lu [1,2,3,*]

[1] Department of Horticulture, College of Agriculture, Shihezi University, Shihezi 832003, China

[2] Key Laboratory of Special Fruits & Vegetables Cultivation Physiology and Germplasm Resources
Utilization of Xinjiang Production and Construction Group, Shihezi 832003, China

[3] Workstation for Academician Li Tianlai, Shihezi University, Shihezi 832003, China

* Correspondence: guozhihua12@126.com (Z.G.); lxyan_agr@shzu.edu.cn (X.L.)

SUPPLEMENTARY FIGURE

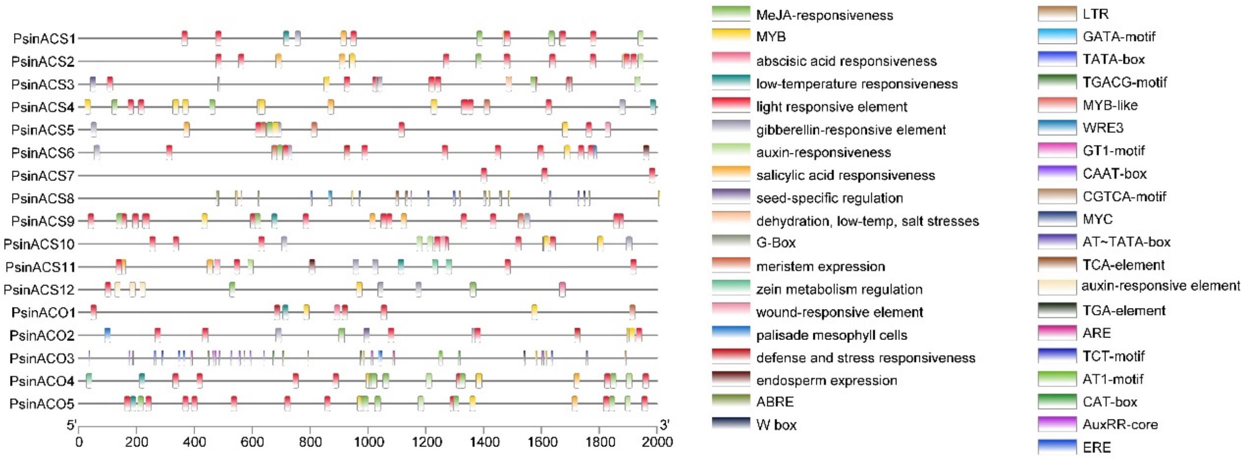

Figure S1 Cis-regulatory element analysis of the promoters of *PsinACS* and *PsinACO* genes in *Pyrus sinkiangensis*.

The upstream promoter regions (2,000 bp) of 12 *PsinACS* and 5 *PsinACO* genes were analyzed by scanning the PlantCARE database. Various cis-acting regulatory elements are represented by different colored boxes, including hormone-responsive elements, stress-responsive elements, transcription factor binding sites, developmentally regulated elements and other regulatory motifs.
